# Assessment of Dental Care Utilization Based on Health Information Literacy in Korean Adults

**DOI:** 10.3390/dj13100467

**Published:** 2025-10-14

**Authors:** Sun-Kyoung Lee, Jeong-Min Seong

**Affiliations:** 1 Department of Dental Technology, College of Biotechnology & Health, Shinhan University, Uijeongbu 11644, Republic of Korea; oksk3737@hanmail.net; 2 Department of Dental Hygiene, Kangwon National University, Samcheok 25949, Republic of Korea

**Keywords:** dental use behavior, emergency, health information literacy, prevention, regular checkup

## Abstract

**Background/Objectives:** This study aimed to examine the utilization of dental care services according to the health information literacy among Korean adults. **Methods:** This study used secondary raw data from the 9th National Health and Nutrition Examination Survey (2023), which included 3356 adults aged 20 years and older. Frequency analysis, cross-analysis, and logistic regression were performed (*p* < 0.05). Data were analyzed using IBM SPSS software (ver. 22.0). **Results:** Using cross-analysis to identify the relationship between health information understanding and oral examinations within the last year, statistically significant differences were confirmed for all questions (*p* < 0.05). A statistically significant association was identified between individuals’ level of health information literacy and their engagement in preventive dental care, particularly the utilization of scaling procedures (*p* < 0.05). The higher their understanding of health information, the more likely they were to have undergone an oral examination in the previous year (nearly twice as likely higher; *p* = 0.003). The higher the understanding of health information regarding preventive treatment (scaling), the more likely the participant was to receive it (i.e., 2.2 times higher; *p* < 0.001). **Conclusions:** Educational interventions and policy support to improve the ability to understand health information can be important strategies for promoting the practice of preventive oral health and improving oral health level in people.

## 1. Introduction

Health information literacy indicates an individual’s complex ability to access, understand, and use health information after judging its accuracy to manage and promote health and prevent health problems [1]. Integrated health information literacy is considered not only a simple ability to learn information but also a multidimensional skill necessary for making health-related decisions [1]. While the most common approach to defining health information literacy considers the ability to read and interpret text-based health information, such as prescriptions [2], the concept is closely associated with various health indicators, such as chronic disease morbidity, utilization of healthcare services, and health behavior [3,4], as well as age, education level, and income level. In the dental health context, health information literacy significantly affects various aspects, such as prevention of caries, maintenance of healthy gums, and decisions regarding when to avail dental care services [5]. Health literacy is a strong predictor of an individual’s health status, behaviors, and outcomes. Low health literacy is associated with reduced use of preventive services, delayed diagnosis, poor adherence to medical instructions, inadequate self-management, increased mortality risk, worse health outcomes, and higher healthcare costs [6].

The World Health Organization prescribes health information literacy as a major factor in determining good health conditions [4,7]. Accordingly, many countries have established policies and strategies to improve health information literacy. Japan and China have set improvement in health information literacy as a major public healthcare project at the national level and have promoted the public’s ability to improve their basic knowledge about health, health management, and lifestyle [8].

Similarly, in terms of dental health, performing proper health-related behavior by learning and understanding health information is regarded as an essential factor for maintaining and promoting oral health [9]. Nevertheless, many adults do not sufficiently understand health information. In the U.S., approximately 36% of adults have less than basic health information literacy [10]. In Korea, 37.1% of adults did not understand patient medication information, and 28.1% of adults had worse health information literacy levels than middle-school students [11].

Oral health is an important factor in maintaining quality of life and overall health status. Good oral health allows people to consume a variety of foods and has a positive impact on pronunciation and social interactions [12]. However, the Korea National Health and Nutrition Examination Survey (KNHANES) shows that many people do not undergo regular dental examinations and tend to postpone receiving dental treatment [13]. This trend is remarkable among those with poor health information literacy. In this population, the proportion of patients who underwent emergency dental treatment was higher than that of patients who underwent preventive dental treatment [14]. Studies using KNHANES data found that people with high health information literacy tended to undergo regular dental examinations and actively use preventive treatment [15]. By contrast, people with poor health information literacy tended to postpone receiving dental treatment and often use emergency treatment, likely leading to poor oral health and quality of life in the long term and high medical cost burdens [16].

Therefore, this study aimed to analyze the effect of health information literacy on dental use behavior based on KNHANES data, which further proposes effective oral health education and policy-level intervention plans. Ultimately, this study aimed to contribute to the promotion of overall oral health by improving the public’s ability to understand health information.

## 2. Materials and Methods

### 2.1. Participants

This study used secondary raw data from the 9th KNHANES for the year 2023 [17]. In 2023, the second year of the 9th KNHANES, 9825 participants were included, of whom 6929 participated in the survey (participation rate of 70.5%). Korean adults were defined as those aged ≥20 years, and of 5866 Korean adults aged ≥20 years, those who did not respond to questions related to dental prosthesis and oral health were excluded. Of the 5644, those classified as ineligible for dental examination were also excluded. Data were collected from the final 3356 participants.

### 2.2. Methods

The survey contained 10 items regarding health information literacy, and each item was rated on a 4-point Likert scale (1 = strongly disagree, 2 = disagree, 3 = agree, and 4 = strongly agree). In this study, “strongly disagree” and “disagree” were categorized as “low,” while “agree” and “strongly agree” were categorized as “high.”

General characteristics, including sex, age, income level, and education level, were evaluated. Dental use behavior was classified into periodic oral examinations and preventive and emergency treatments. Periodic oral examinations comprised oral examination over the last year, while preventive treatments comprised prevention of tooth decay (teeth home filling, fluoride) and scaling. Finally, emergency treatments comprised treatment for tooth decay, extraction, or oral surgery.

In this study, “teeth home filling” was interpreted as preventive resin restorations, a common preventive treatment in Korea for managing early carious lesions. This category was analyzed along with sealants and topical fluoride applications under preventive treatments.

### 2.3. Statistical Analysis

Data were analyzed using SPSS (ver. 22.0, IBM Corp., Armonk, NY, USA). Participants’ general characteristics and dental use behavior were analyzed using descriptive statistics. A cross-analysis was performed to identify the association of health information literacy with oral examination over the last year, preventive dental treatment, and emergency dental treatment. Logistic regression was performed to investigate the effects of health information literacy on dental use behavior. To examine the impact of health information literacy (HIL) on dental care utilization behaviors, logistic regression analysis was performed. The dependent variables included whether the participant had undergone an oral examination within the past year, received preventive treatments (e.g., sealants, topical fluoride application), undergone scaling, or used emergency dental services (e.g., treatment of dental caries, tooth extraction, oral surgery). The primary independent variable was the dichotomized HIL level (“low” vs. “high”), and covariates such as sex, age, education level, and income were included to adjust for potential confounding effects.

Additionally, age and recent experience of oral examination were specifically included as covariates in the regression models to further control for their influence on the relationship between HIL and dental care utilization behaviors. A *p*-value of 0.05 was considered statistically significant.

## 3. Results

### 3.1. Participants’ General Characteristics

This study analyzed data from 3356 adults aged ≥20 years; Table 1 reports the general characteristics. Of the adults, 56.9% were women, and 43.1% were men. The age group of 61–70 years accounted for the highest proportion (24.7%), followed by the age groups of 51–60 years (20.6%), ≥71 years (16.8%), 41–50 years (15.8%), 31–40 years (11.4%), and 20–30 years (10.8%). The proportions of participants with high-, upper-middle-, lower-middle-, and low-income levels were 28.7%, 25.6%, 24.0%, and 21.7%, respectively. Participants with a college or higher education level (42.0%) accounted for the highest proportion concerning education level, followed by high school graduates (32.4%), elementary school education or lower (14.8%), and middle-school graduates (10.8%).

Regarding dental use behavior (periodic examination, prevention, and emergency treatment), 68.2% had undergone oral examinations over the last year, while 31.8% had not. Among the preventive treatments, 6.1% received treatment to prevent tooth decay (teeth home filling, fluoride) and 70.9% received scaling. Most respondents received preventive treatments such as scaling. The proportion of respondents who underwent emergency treatment was relatively low, with 19.8% receiving treatment for tooth decay and 15.9% undergoing extraction or oral surgery.

### 3.2. Relationship Between Health Information Literacy and Oral Examination in the Last Year

A cross-analysis was performed to determine the association between health information literacy and oral examinations in the last year, revealing a statistically significant difference for every item (*p* < 0.05).

For the question, “Can you determine the necessary vaccinations?,” the proportion of respondents who underwent oral examinations was 55.5% in the group with high health information literacy and 12.7% in the group with low health information literacy (*p* = 0.000). In other words, a higher cognitive level regarding prevention-related information indicated a significantly higher participation rate in oral examinations. For the question, “Can you understand the degree of risks that occur due to mental health problems such as stress and depression?,” the proportion of respondents who received oral examinations was 55.5% in the group with a high level of understanding of mental health-related information and 12.8% in the group demonstrating low literacy (*p* = 0.004). For the question, “Do you know what health signals can occur due to smoking, too much work, and lack of exercise?,” the proportion of respondents who underwent oral examinations was 60.0% in the group that understood the risks of a bad lifestyle and 8.2% in the group demonstrating low literacy (*p* = 0.000). For the question “Can you determine which of your daily life activities affects your health?,” the proportion of respondents who received oral examinations was 61.5% in the group with a high ability to determine influential factors on health and 6.7% in the group demonstrating low literacy (*p* = 0.000). For the question, “Can you understand the doctor’s explanation and instructions when getting treatment?” The proportion of respondents who underwent oral examinations was 65.3% in the high communication skills group and 2.9% in the low communication skills group (*p* = 0.003). For the question, “Can you determine what to do first when there is an emergency?,” the proportion of respondents who received oral examinations related to problem solving was 59.7% in the group with high health information literacy and 8.5% in the group with low literacy (*p* = 0.002). For the question, “Can you understand how to take medicine explained by a doctor or pharmacist?,” the proportion of respondents who received oral examinations was 66.6% in the group with a high ability to understand patient dosing instructions and 1.6% in the group with low ability (*p* = 0.023). For the question, “Do you understand the educational materials patients receive at the hospital?,” the proportion of respondents who underwent oral examinations was 62.0% in the group with a high level of understanding of health-related educational materials and 6.0% in the group with a low level (*p* = 0.000). For the question “Can you determine if the health information obtained from the Internet or the media is reliable?,” the proportion of respondents who received oral examinations was 54.7% in the group with a high level of critical determination and 13.5% in the group with a low level (*p* = 0.000). For the question, “Can you use the health information obtained from the Internet or the media for health-related behavior or decision?,” the proportion of respondents who underwent oral examinations was 56.0% in the group with a high ability to convert information to behavior and 12.2% in the group with low ability. There were significant differences between the groups (*p* = 0.000) (Table 2).

### 3.3. Relationship Between Health Information Literacy and the Use of Preventive Dental Care

Table 3 presents the results of chi-square testing for the relationship between health information literacy and the use of preventive dental care. A distinct difference was observed in the use of dental scaling according to the level of health information literacy.

For the question, “Can you determine the necessary vaccinations?,” the proportion of respondents who underwent dental scaling was 57.5% in the group with high health information literacy and 13.4% in the group with poor literacy (*p* = 0.000). For the question, “Can you understand the degree of risks that occur due to mental health problems?,” the proportion of respondents who underwent dental scaling was 58.2% in the high-literacy group and 12.7% in the low-literacy group (*p* = 0.000). For the question, “Do you know what health signals can occur due to smoking, too much work, and lack of exercise?,” the proportion of respondents who underwent dental scaling was 31.9% in the high-literacy group and 9.0% in the low-literacy group (*p* = 0.000). For the question, “Can you determine which of your daily life activities affects your health?,” the proportion of respondents who underwent dental scaling was 63.5% in the high-literacy group and 7.4% in the low-literacy group (*p* = 0.000). For the question, “Can you understand the doctor’s explanation and instructions when receiving treatment?,” the proportion of respondents who underwent dental scaling was 68.4% in the high-literacy group and 2.5% in the low-literacy group (*p* = 0.000). For the question, “Can you determine what to do first when there is an emergency?,” the proportion of respondents who underwent dental scaling was 62.0% in the high-literacy group and 8.9% in the low-literacy group (*p* = 0.000). For the question, “Do you understand how to take medicine as explained by a doctor or pharmacist?,” the proportion of respondents who underwent dental scaling was 69.4% in the high-literacy group and 1.5% in the group with low ability (*p* = 0.000). For the question, “Do you understand the educational materials patients receive at the hospital?’ The proportion of respondents who underwent dental scaling was 64.9% in the high-literacy group and 6.0% in the low-literacy group (*p* = 0.000). For the question “Can you determine if the health information obtained from the media is reliable?” The proportion of respondents who underwent dental scaling was 56.0% in the high-literacy group and 14.9 in the low-literacy group (*p* = 0.006). For the question, “Can you use the health information obtained from the Internet or the media for health-related behavior or decision?” The proportion of respondents who underwent dental scaling was 58.3% in the high-literacy group and 12.6% in the low-literacy group (*p* = 0.000).

A significant difference was also observed in the use of preventive care for tooth decay between the two questions: “Can you determine if the health information obtained from the Internet or the media is reliable?” and “Can you use the health information obtained from the Internet or the media for health-related behavior or decision?” In particular, preventive behavior related to tooth decay was associated with the evaluation of the reliability of health information from the Internet (*p* = 0.008) and the ability to use health information (*p* = 0.024).

### 3.4. Relationship Between Health Information Literacy and the Use of Emergency Treatment in Dentistry

The relationship between the health information literacy and emergency dentistry use is shown in Table 4. Regarding the question, “do you know what a health signal that can occur due to smoking, too much work, and lack of exercise?” There was a significant difference in the experience of tooth extraction between those with high and those with poor literacy (*p* = 0.032).

The proportion of respondents who underwent tooth extraction was 12.2% in the group with high ability to identify signals of health risk and 18.9% in the group with poor ability. Regarding the question “Can you determine which of your daily life affects your health?” There was a significant difference in the experience of tooth extraction between those with high and those with poor literacy (*p* = 0.019).

### 3.5. Impact of Health Information Literacy on Behavior Regarding Dental Treatment Use

Table 5 presents the results of evaluating the impact of health information literacy on dental treatment use behavior. With regard to regular oral examinations (over the last year), the respondents who had higher health information literacy had approximately 1.815 times {B = 0.596, Exp (B) = 1.815} higher likelihood of receiving oral examinations in the following year. This result is statistically significant (*p* = 0.003) and implies that higher health information literacy indicates a greater likelihood of undergoing regular checkups. Regarding preventive treatment (dental scaling), the respondents with higher health information literacy had an approximately 2.251 times {B = 0.811, Exp (B) = 2.251} higher likelihood of receiving dental scaling. This result was statistically significant (*p* < 0.001) and implied that higher health information literacy indicated a greater likelihood of actively receiving preventive dental treatment. Regarding emergency treatment (tooth extraction), the level of health information literacy did not significantly impact the experience of tooth extraction (*p* > 0.05).

## 4. Discussion

This study used the 9th KNHANES data to analyze the effects of health information literacy on dental use behavior, revealing a statistically significant rate of receiving regular oral examinations and using preventive dental care, such as scaling, in the group with high health information literacy. However, no significant association was observed between health information literacy and tooth extraction, which is an emergency treatment item. This implies that health information literacy closely affects the practice behavior of oral health, especially the use of prevention-centered dental care. Health literacy has been found to be a strong predictor of an individuals’ health, health behavior and health outcomes [3,18]. Limited health literacy is associated with poor self-ratings of health, poor adherence to medical instructions, poor self-management skills, increased mortality risks, poor health outcomes, and higher healthcare costs [19,20,21].

Regression analysis of items among regular oral examinations showed that respondents with higher health information literacy had an approximately 1.8 times higher likelihood of receiving oral examinations and an approximately 2.25 times higher likelihood of undergoing dental scaling, showing much more distinct aspects. Kim [15] reported that individuals with higher health information literacy tended to actively undergo regular dental checkups and preventive dental services. Consistent results were observed in this study, confirming a causal relationship between the ability to understand dental health information and applying dental health-related behaviors.

No statistically significant difference was found in tooth extraction during emergency treatment between patients with high and poor health information literacy. Individuals with high information literacy are more likely to take preventive measures. However, we believe that emergency treatments such as tooth extraction are likely to be affected by various external factors such as pain, insufficient time, and accessibility problems. However, a group with poor health information literacy can miss the time for treatment owing to a lack of regular management, ultimately resulting in emergency treatment. Ju et al. [14] reported that the proportion of individuals who relied on emergency treatment was higher in a group of adults with poor ability to understand dental health information and mentioned that the number of dental care visits in the group was irregular, which is consistent with the results of this study.

The relationship between health information literacy and dental use behavior was analyzed. Dental use behavior was significantly associated with overall levels of health information literacy or, in other words, understanding the lifestyle that affects health, understanding the instructions of healthcare professionals, and the ability to determine the reliability of digital information and applying it. This emphasizes the importance of the simple ability to acquire information and ensuring comprehensive literacy, which includes the ability to think critically and take health-related actions based on critical thinking. According to Hong et al. [22], children in upper elementary schools with high oral health literacy showed better brushing techniques and a higher rate of participation in dental checkups. Similar patterns were observed in studies on adults [23,24,25,26,27]. Lee et al. [16] suggested that a Korean version of the oral health literacy assessment tool should be developed to measure the ability to use and apply information in practice beyond simple reading and understanding. The group of respondents who answered that they could use health information showed a high rate of undergoing preventive dental treatments. This implies that an improved understanding of information can lead to practical health-related behaviors.

Based on these findings, we conclude with the following policy suggestions. First, oral health education for the public should be modified to include health literacy-based education that emphasizes not only simple information transmission but also the interpretation and application of such information. Second, health information literacy should be evaluated for various age groups and social classes, and customized educational programs are required. Education for adolescents and young adults who do not have problems using digital information should be distinguished from education for older adults and vulnerable groups with poor accessibility to digital information. Third, medical institutes and public health centers should provide understandable health information through a patient-centered communication approach, in combination with consultation and motivation programs for the public. In future studies, biology- or diagnosis-based analyses are necessary to investigate the association between health information literacy and actual oral health conditions (decay index, periodontal diseases, etc.). Future studies with a longitudinal design should demonstrate the extent to which preventive behaviors reduce the incidence of emergencies. Moreover, an experimental study on the educational effects of health literacy improvement programs should be conducted, which can be used as evidence to establish specific public policies.

This study has several limitations that should be acknowledged. First, although the dichotomization of HIL scores facilitated analysis, it may have reduced data variability and limited the interpretation of nuanced trends. Second, multiple chi-square tests were performed without statistical correction, which may increase the risk of type I error. Third, effect sizes were not reported, hindering the assessment of practical significance. In addition, as only scaling was widely reported among preventive treatments, generalizability to other forms of care remains limited. Given the cross-sectional nature of this study, causal relationships cannot be inferred. Furthermore, unmeasured confounding variables—such as dental insurance status, previous negative experiences, or dental anxiety—may have influenced dental behavior. Fourth, the use of self-reported data may be subject to recall or social desirability bias. These limitations should be addressed in future longitudinal and multi-factorial studies. Finally, age may be associated with digital literacy and access to online health information, which in turn can affect the level of health information literacy. This potential confounding effect of age should be considered when interpreting the results. These limitations should be addressed in future longitudinal and multi-factorial studies.

## 5. Conclusions

This study analyzed the effects of health information literacy on dental use behavior and showed that individuals with high health information literacy tended to actively use preventive dental treatments, such as regular oral examinations and scaling. However, a high proportion of those with poor health information literacy postponed dental treatment and heavily relied on emergency treatment. This indicates that health information literacy mainly affects individuals’ oral health behaviors. Therefore, interventions and policy support to improve the ability to understand health information will promote the practice of prevention-centered oral healthcare and can be an important strategy for improving the public’s overall oral health conditions. Longitudinal research should be conducted to develop a program to improve customized health literacy for various groups of people and evaluate its long-term effects.

## Figures and Tables

**Table 1:** General characteristics and dental use behavior; n (%): 3356 (100.0).

Characteristics		Category	n	Percentage
Sex		Male	1447	43.1
		Female	1909	56.9
Age		20–30	361	10.8
		31–40	383	11.4
		41–50	529	15.8
		51–60	690	20.6
		61–70	830	24.7
		71 years old or older	563	16.8
Income level		Low	729	21.7
		Lower-middle	805	24.0
		Upper-middle	860	25.6
		High	962	28.7
Education level		≥Elementary school	496	14.8
		Middle school	363	10.8
		High school	1086	32.4
		College≤	1409	42.0
Dental use behavior				
Examination	Oral examination over the last year	Yes	2289	68.2
		No	1067	31.8
Prevention treatment	Prevention of tooth decay (teeth home filling, fluoride)	Yes	204	6.1
		No	3152	93.9
	Scaling	Yes	2380	70.9
		No	976	29.1
Emergency treatment	Caries	Yes	669	19.8
		No	2691	80.2
	Extraction or oral surgery	Yes	526	15.7
		No	2830	84.3

**Table 2:** Relationship between health information literacy and oral examination in the last year; n (%) = 3356 (100.0).

Health Information Literacy		Oral Examination in the Last Year		
		No	Yes	*p*
1. Can you determine the necessary vaccinations?	Low	269 (8.0)	425 (12.7)	0.000
	High	798 (23.8)	1864 (55.5)	
2. Can you understand the degree of risks that occur due to mental health problems?	Low	245 (7.3)	428 (12.8)	0.004
	High	822 (24.5)	1861 (55.5)	
3. Do you know what health signals can occur due to smoking, too much work, and lack of exercise?	Low	229 (6.8)	274 (8.2)	0.000
	High	838 (25.0)	2015 (60.0)	
4. Can you determine which of your daily life activities affects your health?	Low	187 (5.6)	226 (6.7)	0.000
	High	880 (26.2)	2063 (61.5)	
5. Can you understand the doctor’s explanation and instructions when receiving treatment?	Low	70 (2.1)	96 (2.9)	0.003
	High	997 (29.7)	2193 (65.3)	
6. Can you determine what to do first when there is an emergency?	Low	174 (5.2)	284 (8.5)	0.002
	High	893 (26.6)	2005 (59.7)	
7. Do you understand how to take medicines as explained by a doctor or pharmacist?	Low	40 (1.2)	54 (1.6)	0.023
	High	1027 (30.6)	2235 (66.6)	
8. Do you understand the educational materials patients receive at the hospital?	Low	140 (4.2)	201 (6.0)	0.000
	High	927 (27.6)	2088 (62.2)	
9. Can you determine if the health information obtained from the Internet or the media is reliable?	Low	293 (8.7)	453 (13.5)	0.000
	High	774 (23.1)	1836 (54.7)	
10. Can you use the health information obtained from the Internet or the media for health-related behavior or decision?	Low	273 (8.1)	411 (12.2)	0.000
	High	794 (23.7)	1878 (56.0)	

Analysis conducted using Pearson’s chi-square test.

**Table 3:** Relationship between health information literacy and the use of preventive dental care n (%) = 3356 (100.0).

Health Information Literacy		Fluoride		*p*	Scaling		
		No	Yes		No	Yes	*p*
1. Can you determine the necessary vaccinations?	Low	655 (19.5)	39 (1.2)	0.570	244 (7.3)	450 (13.4)	0.000
	High	2497 (74.4)	165 (4.9)		732 (21.8)	1930 (57.5)	
2. Can you understand the degree of risks that occur due to mental health problems?	Low	641 (19.1)	32 (1.0)	0.108	247 (7.4)	426 (12.7)	0.000
	High	2511 (74.8)	172 (5.1)		729 (21.7)	1954 (58.2)	
3. Do you know what health signals can occur due to smoking, too much work, and lack of exercise?	Low	473 (14.1)	30 (0.9)	0.907	202 (6.0)	301 (9.0)	0.000
	High	2679 (79.8)	174 (5.2)		774 (23.1)	2079 (31.9)	
4. Can you determine which of your daily life activities affects your health?	Low	391 (11.7)	22 (0.7)	0.495	164 (4.9)	249 (7.4)	0.000
	High	2761 (82.3)	182 (5.4)		812 (24.2)	2131 (63.5)	
5. Can you understand the doctor’s explanation and instructions when receiving treatment?	Low	160 (4.8)	6 (0.2)	0.173	81 (2.4)	85 (2.5)	0.000
	High	2992 (89.2)	198 (5.9)		895 (26.7)	2295 (68.4)	
6. Can you determine what to do first when there is an emergency?	Low	433 (12.9)	25 (0.7)	0.550	158 (4.7)	300 (8.9)	0.000
	High	2719 (81.0)	179 (5.3)		818 (24.4)	2080 (62.0)	
7. Do you understand how to take medicines as explained by a doctor or pharmacist?	Low	86 (2.6)	8 (0.2)	0.317	43 (1.3)	51 (1.5)	0.000
	High	3066 (91.4)	196 (5.8)		933 (27.8)	2329 (69.4)	
8. Do you understand the educational materials patients receive at the hospital?	Low	326 (9.7)	15 (0.4)	0.171	140 (4.2)	201 (6.0)	0.000
	High	2826 (84.2)	189 (5.6)		836 (24.9)	2179 (64.9)	
9. Can you determine if the health information obtained from the Internet or the media is reliable?	Low	716 (21.3)	30 (0.9)	0.008	247 (7.4)	499 (14.9)	0.006
	High	2436 (72.6)	174 (5.2)		729 (21.7)	1881 (56.0)	
10. Can you use the health information obtained from the Internet or the media for health-related behavior or decision?	Low	655 (19.5)	29 (0.9)	0.024	260 (7.7)	424 (12.6)	0.000
	High	2497 (74.4)	175 (5.2)		716 (21.3)	1956 (58.3)	

Analysis conducted using Pearson’s chi-square test.

**Table 4:** Relationship between health information literacy and the use of emergency treatment in dentistry; n (%) = 3356 (100.0).

Health Information Literacy		Caries			Extraction		
		No	Yes	*p*	No	Yes	*p*
1. Can you determine the necessary vaccinations?	Low	540 (16.1)	154 (4.6)	0.078	584 (17.4)	110 (3.3)	0.886
	High	2151 (64.1)	511 (15.2)		2246 (66.9)	416 (12.4)	
2. Can you understand the degree of risks that occur due to mental health problems?	Low	554 (16.5)	119 (3.5)	0.120	558 (16.6)	115 (3.4)	0.259
	High	2137 (63.7)	546 (16.3)		2272 (67.7)	411 (12.2)	
3. Do you know what health signals can occur due to smoking, too much work, and lack of exercise?	Low	414 (12.3)	89 (2.7)	0.195	408 (12.2)	95 (2.8)	0.032
	High	2277 (67.8)	576 (17.2)		2422 (72.2)	431 (12.8)	
4. Can you determine which of your daily life activities affects your health?	Low	338 (10.1)	75 (2.2)	0.367	332 (9.9)	81 (2.4)	0.019
	High	2353 (70.1)	590 (17.6)		2498 (74.4)	445 (13.3)	
5. Can you understand the doctor’s explanation and instructions when receiving treatment?	Low	131 (3.9)	35 (1.0)	0.674	139 (4.1)	27 (0.8)	0.830
	High	2560 (76.3)	630 (18.8)		2691 (80.2)	499 (14.9)	
6. Can you determine what to do first when there is an emergency?	Low	354 (10.5)	104 (3.1)	0.095	379 (11.3)	79 (2.4)	0.318
	High	2337 (69.6)	561 (16.7)		2451 (73.0)	447 (13.3)	
7. Do you understand how to take medicines as explained by a doctor or pharmacist?	Low	76 (2.3)	18 (0.5)	0.869	76 (2.3)	18 (0.5)	0.347
	High	2615 (77.9)	647 (19.3)		2754 (82.1)	508 (15.1)	
8. Do you understand the educational materials patients receive at the hospital?	Low	278 (8.3)	63 (1.9)	0.512	284 (8.5)	57 (1.7)	0.577
	High	2413 (71.9)	602 (17.9)		2546 (75.9)	469 (14.0)	
9. Can you determine if the health information obtained from the Internet or the media is reliable?	Low	589 (17.6)	157 (4.7)	0.339	619 (18.4)	127 (3.8)	0.250
	High	2102 (62.6)	508 (15.1)		2211 (65.9)	399 (11.9)	
10. Can you use the health information obtained from the Internet or the media for health-related behavior or decision?	Low	555 (16.5)	129 (3.8)	0.482	566 (16.9)	118 (3.5)	0.203
	High	2136 (63.6)	536 (16.0)		2264 (67.5)	408 (12.2)	

Analysis conducted using Pearson’s chi-square test.

**Table 5:** Impact of health information literacy on behavior regarding dental treatment use; n (%) = 3356 (100.0).

	Variable	Regression (B)	Standard Error (SE)	Exp (B)	*p*
Examination	Oral examination in the last year	0.596	0.198	1.815	0.003
Preventive treatment	Scaling	0.811	0.196	2.251	0.000
Emergency treatment	Extraction	0.015	0.255	1.015	0.953

Analysis conducted using logistics regression.

## Data Availability

The original data presented in the study are openly available in https://knhanes.kdca.go.kr/knhanes/postSendPage.do?url=/rawDataDwnld/rawDataDwnld.do&postparam=%7B%22menuId%22:%2210031001%22%7D, accessed on 30 April 2025.

## Abbreviations

The following abbreviations are used in this manuscript:

KNHANES	Korea National Health and Nutrition Examination Survey
